# Genome-wide detection of CNV regions between Anqing six-end-white and Duroc pigs

**DOI:** 10.1186/s13039-023-00646-0

**Published:** 2023-07-03

**Authors:** Rong Qian, Fei Xie, Wei Zhang, JuanJuan Kong, Xueli Zhou, Chonglong Wang, Xiaojin Li

**Affiliations:** 1 Institue of Agricultural Economics and Information, Anhui Academy of Agricultural Sciences, Hefei, 230031 Anhui China; 2 Institue of Animal Husbandry and Veterinary Medicine, Anhui Academy of Agricultural Sciences, Hefei, 230031 Anhui China; 3 College of Animal Science, Anhui Science and Technology University, Fengyang County, 233100 Anhui Province China

**Keywords:** Copy number variation, Genome-wide, Anqing six-end-white pigs, Duroc pigs

## Abstract

**Background:**

Anqing six-end-white pig is a native breed in Anhui Province. The pigs have the disadvantages of a slow growth rate, low proportion of lean meat, and thick back fat, but feature the advantages of strong stress resistance and excellent meat quality. Duroc pig is an introduced pig breed with a fast growth rate and high proportion of lean meat. With the latter breed featuring superior growth characteristics but inferior meat quality traits, the underlying molecular mechanism that causes these phenotypic differences between Chinese and foreign pigs is still unclear.

**Results:**

In this study, copy number variation (CNV) detection was performed using the re-sequencing data of Anqing Six-end-white pigs and Duroc pigs, A total of 65,701 CNVs were obtained. After merging the CNVs with overlapping genomic positions, 881 CNV regions (CNVRs) were obtained. Based on the obtained CNVR information combined with their positions on the 18 chromosomes, a whole-genome map of the pig CNVs was drawn. GO analysis of the genes in the CNVRs showed that they were primarily involved in the cellular processes of proliferation, differentiation, and adhesion, and primarily involved in the biological processes of fat metabolism, reproductive traits, and immune processes.

**Conclusion:**

The difference analysis of the CNVs between the Chinese and foreign pig breeds showed that the CNV of the Anqing six-end-white pig genome was higher than that of the introduced pig breed Duroc. Six genes related to fat metabolism, reproductive performance, and stress resistance were found in genome-wide CNVRs (*DPF3*, *LEPR*, *MAP2K6*, *PPARA*, *TRAF6*, *NLRP4*).

## Background

The study of genetic variation is an important prerequisite and basis for exploring the characteristics of pig breeds and implementing breeding programs based on molecular technologies. Anqing six-end-white (AQSW) pig is a protected national livestock breed that features the advantages of early sexual maturity, strong disease resistance, high fertility, and excellent meat quality, among other desirable characteristics. Duroc is a major commercial pig breed known for its fast growth rate and high proportion of lean meat [1]. There are substantial differences in the growth, meat quality, and disease resistance between these two pig breeds; the former being Chinese in origin and the latter being foreign in origin [2]. However, little is known about the genetic basis responsible for the phenotypic differences between such representative Chinese and foreign pig breeds. Copy number variation (CNV) is an important source of genetic variation that can be used to identify genetic variation among breeds. CNV mainly affects gene expression and function, and it does so through multiple mechanisms, such as altering gene dosage, disrupting coding sequences, and perturbing the long-range regulation of genes. CNVs are considered to be another important contributor to genetic variation other than SNPs [3].

CNV represents an important component of structural variation within the genome. Genome structural variation is closely related to phenotypic diversity and human disease, but the mechanisms explaining its functional impact have remained elusive. CNV is one aspect of genomic variation that is present in a variety of organisms and acts on different molecular and cellular processes, thereby resulting in a variety of changes in traits such as disease susceptibility, phenotypic diversity, and evolutionary differences. Some CNVRs that affect important economic traits in pigs have been successfully identified. For example, Wang [4] conducted a genome-wide association analysis between CNVs and meat quality traits in pigs and identified 8 CNVRs that were significantly associated with at least one meat quality trait. A genome-wide association analysis of porcine CNVRs based on next-generation sequencing data revealed that CNVRs were associated with genetic variation in fatty acid composition and growth traits [5]. A genome-wide comparison of CNVs in Tongcheng pigs and large white pigs using genome sequencing data showed that two genes, DCUN1D2 and SPARCL1, were associated with disease resistance in pigs, while PLEKHA2 and SLCO1A2 were found to be related to lipid metabolism [6]. The CNVs of Meishan pigs and Duroc pigs were studied using next-generation sequencing technology to discover that the copy number of AHR had a positive effect on pig litter size (*P* < 0.05) [7]. Qiu et al. [8] found that four CNVRs were significantly correlated with both average daily weight gain and net weight gain, thereby suggesting that these CNVRs play an important role in regulating growth and fat deposition in pigs.

In past studies, it has been recognized that a major driver of variation among individuals is variation in the copy number of genomic segments and the genes contained within those segments, and such CNVs have evolved in species other than primates [9–12]. AQSW pig is a local pig in Anhui Province,which has the disadvantages of a slow growth rate, low proportion of lean meat.

## Results

### Genome-wide detection of CNVs

We found a total of 25,463 CNVs in the 20 LB samples, including 13,413 deletions and 12,050 insertions, with an average of 1273 CNVs per pig, and the average size of each CNV was 88,303 bp. Detailed information is shown in Tables 1 and 2.

**Table 1:** Summary of copy number variants (CNVs) of the 20 analysed AQSW pigs

Sample	Number of deletion	Number of duplication	Number of CNVs	Length_avg (bp)	Length_min (bp)	Length_max (bp)
LB1	660	582	1242	122,468	1100	16,700,000
LB2	658	669	1327	113,532	1100	18,300,000
LB3	538	605	1143	156,402	1100	121,000,000
LB4	567	657	1224	122,625	1100	14,100,000
LB5	591	585	1176	130,464	1100	18,200,000
LB6	555	665	1220	123,020	1100	30,800,000
LB7	640	611	1251	118,972	1100	20,500,000
LB8	572	524	1096	148,484	1200	63,600,000
LB9	650	610	1260	117,312	1100	13,100,000
LB10	621	706	1327	112,651	1100	13,300,000
LB11	681	671	1352	71,830	1100	14,500,000
LB12	739	615	1354	46,305	1100	14,400,000
LB13	776	570	1346	45,779	1100	14,500,000
LB14	731	512	1243	50,190	1100	14,500,000
LB15	752	586	1338	47,927	1000	14,500,000
LB16	800	571	1371	44,872	1000	15,600,000
LB17	784	580	1364	46,865	1000	14,500,000
LB18	688	601	1289	51,236	1100	14,500,000
LB19	610	586	1196	51,563	1100	14,500,000
LB20	800	544	1344	43,571	1000	14,500,000

**Table 2:** Summary of copy number variants (CNVs) of the 20 analysed Duroc pigs

Sample	Number of deletion	Number of duplication	Number of CNVs	Length_avg (bp)	Length_min (bp)	Length_max (bp)
Pig_SRX2627693	745	677	1422	7,250,550	1100	14,500,000.00
Pig_SRX2634061	717	800	1517	7,200,550	1100	14,400,000.00
Pig_SRX2634062	797	654	1451	7,250,550	1100	14,500,000.00
Pig_SRX2634815	722	808	1530	7,250,550	1100	14,500,000.00
Pig_SRX2634816	743	686	1429	7,250,550	1100	14,500,000.00
Pig_SRX2635016	694	899	1593	7,250,550	1100	14,500,000.00
Pig_SRX2635040	739	1124	1863	7,250,550	1100	14,500,000.00
Pig_SRX2635041	727	1325	2052	7,250,550	1100	14,500,000.00
Pig_SRX2635046	604	1218	1822	7,250,550	1100	14,500,000.00
Pig_SRX2635047	804	850	1654	7,250,550	1100	14,500,000.00
Pig_SRX2635176	1933	1728	3661	7,250,500	1000	14,500,000.00
Pig_SRX2635177	2062	1753	3815	7,250,550	1100	14,500,000.00
Pig_SRX2637963	717	1145	1862	7,200,550	1100	14,400,000.00
Pig_SRX2645834	658	1845	2503	7,250,550	1100	14,500,000.00
Pig_SRX2648133	723	854	1577	7,250,550	1100	14,500,000.00
Pig_SRX2648644	961	1066	2027	7,800,500	1000	15,600,000.00
Pig_SRX2648645	989	862	1851	8,100,550	1100	16,200,000.00
Pig_SRX2653643	1005	1125	2130	5,300,550	1100	10,600,000.00
Pig_SRX2653645	1370	1092	2462	7,750,550	1100	15,500,000.00
Pig_SRX4063407	634	1383	2017	7,250,550	1100	14,500,000.00

### Differences between CNVRs in Chinese and foreign pig breeds

As determined by the statistics conducted on the CNVRs detected in the LB pig and Duroc pig breeds, it was found that the number of CNVRs detected in LB pigs (an Anqing local pig in Anhui Province) was the largest (461), while the number of CNVRs detected in the introduced Duroc pig breed was lower (421). A possible reason for this result is that introduced pig breeds may have been subjected to strong artificial selection during the breeding process, thereby resulting in such breeds becoming more homozygous and their genetic variation becoming gradually reduced, while local pig breeds in China have been relatively less artificially selected such that their degree of genetic variation is higher than that of introduced pig breeds.

### Comparative analysis of CNVRs in AQSW

**Fig. 1:**
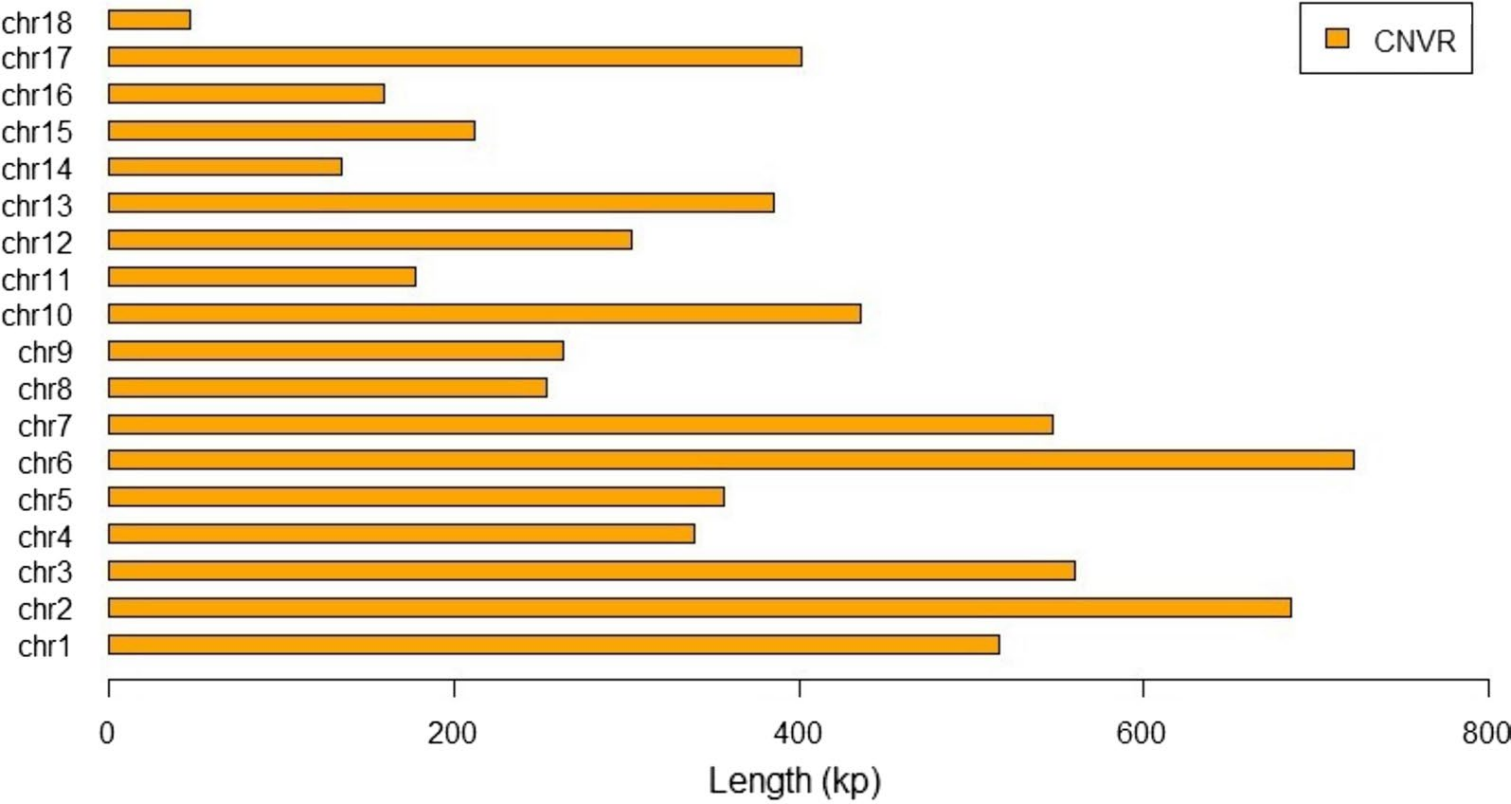
Histogram for the chromosome-wise distribution of 461 CNVRs across the AQSW pig genome

We used BEDTools to merge all CNVs within the AQSW breed to identify 461 CNVRs. As shown in Tables 3 and 4, there were 59 CNVRs on chr2, representing the largest number of CNVRs, and only 4 CNVRs on chr18, representing the least, which were the most common CNVRs across all chromosomes. The length distribution map of the CNVRs is shown in Fig. 1.

**Table 3:** Summary of CNV regions (CNVRs) of AQSW pig breed stratified by chromosome

Chromosome	Number of CNVRs_	Length_Min (bp)	Length_Max (bp)	Length_Avg (bp)	Percentage length in CNVR (%)
chr1	37	599	160,099	13,947	7.9
chr2	59	799	49,899	11,612	10.5
chr3	41	1299	69,299	13,667	8.6
chr4	21	399	82,299	16,141	5.2
chr5	36	2099	58,599	9890	5.5
chr6	39	1299	103,799	18,499	11.1
chr7	38	199	71,599	14,385	8.4
chr8	13	2399	66,499	19,506	3.9
chr9	29	399	30,999	9071	4.0
chr10	29	999	49,899	15,033	6.7
chr11	12	1199	61,199	14,807	2.7
chr12	18	299	102,499	16,849	4.7
chr13	22	399	99,799	17,526	5.9
chr14	14	399	26,699	9613	2.1
chr15	12	2699	57,599	17,640	3.3
chr16	10	4399	51,899	15,919	2.4
chr17	27	899	58,499	14,869	6.2
chr18	4	999	30,099	11,749	0.7

**Table 4:** Summary of CNV regions (CNVRs) of Duroc pig breed stratified by chromosome

Chromosome	Number of CNVRs	Length_Min (bp)	Length_Max (bp)	Length_Avg (bp)	Percentage length in CNVR (%)
chr1	34	299	40,999	9884	5.5
chr2	53	299	71,499	15,022	13
chr3	32	2099	45,999	12,958	6.8
chr4	25	99	40,099	8723	3.6
chr5	28	99	78,399	15,106	6.9
chr6	32	99	99,899	19,077	10
chr7	47	99	49,999	10,522	8.1
chr8	9	6599	55,899	19,199	2.8
chr9	21	199	35,799	11,242	3.8
chr10	22	1899	74,199	28,949	10.5
chr11	11	1399	61,199	12,635	2.3
chr12	23	199	102,499	14,372	5.4
chr13	21	599	99,199	15,099	5.2
chr14	11	2199	23,899	13,154	2.4
chr15	14	1899	56,999	17,263	4.0
chr16	8	4399	47,899	20,374	2.7
chr17	25	2399	53,299	13,547	5.6
chr18	4	499	38,799	18,874	1.2

### Functional annotation of genes

**Fig. 2:**
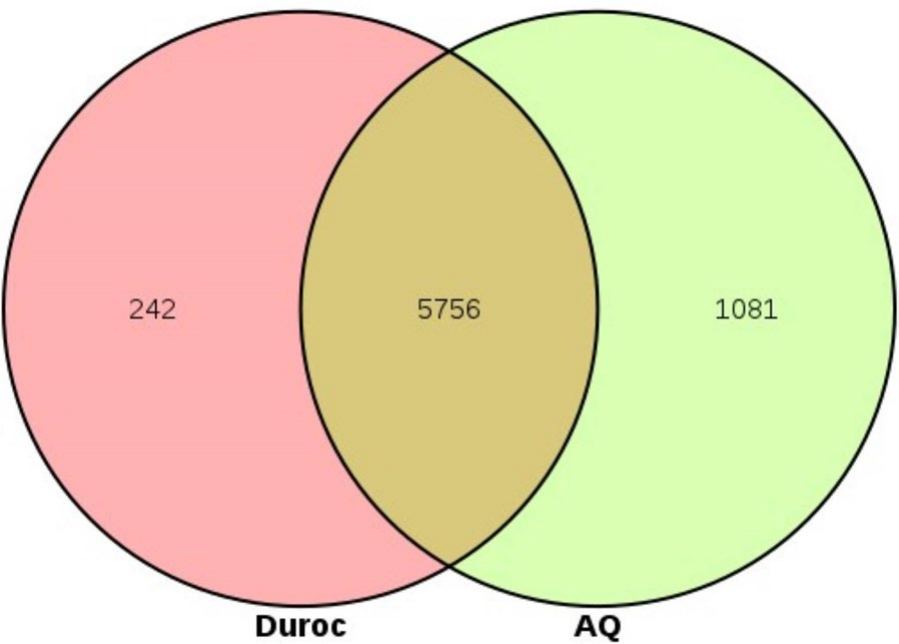
A Venn diagram of respective_genes and common_genes

We detected a total of 15,469 genes with variation between the two pig breeds, among which, 1081 genes were identified in AQSW pigs while 242 genes were identified in Duroc pigs, comprising a total of 5756 genes. These genetic variations may be related to differences in the native environment of the breeds. Adaptability, immunity, and disease resistance are interrelated biological traits that provide a valuable basis for research on the growth and disease susceptibility of pigs. The gene distribution diagram is shown in Fig. 2.

### Enrichment analysis

**Fig. 3:**
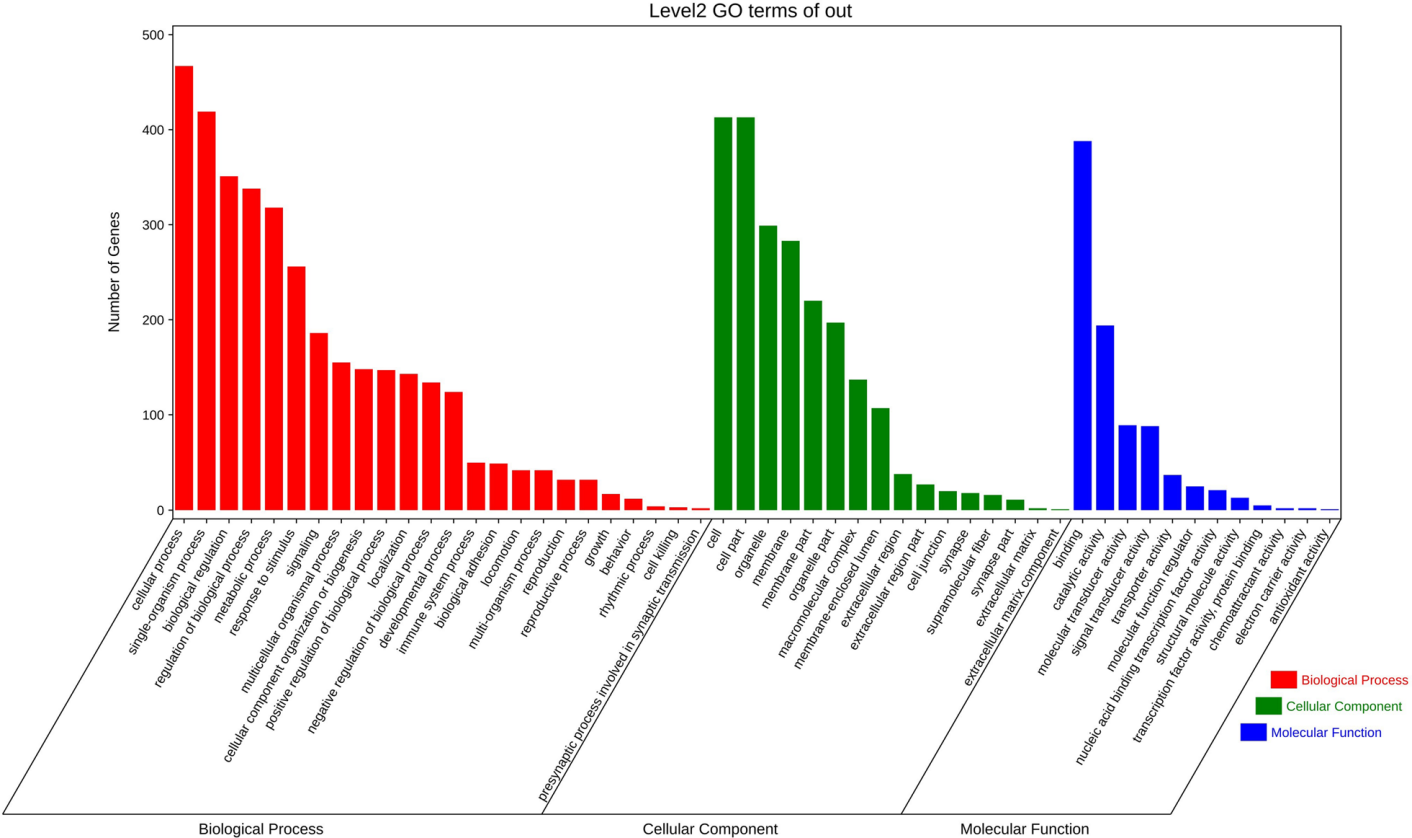
Gene Ontology (GO) terms of the selected genes, red referring to biological process, green referring to cellular component and blue referring to molecular function. *Note*: The horizontal axis represents the enriched items, while the vertical axis represents number of genes

**Fig. 4:**
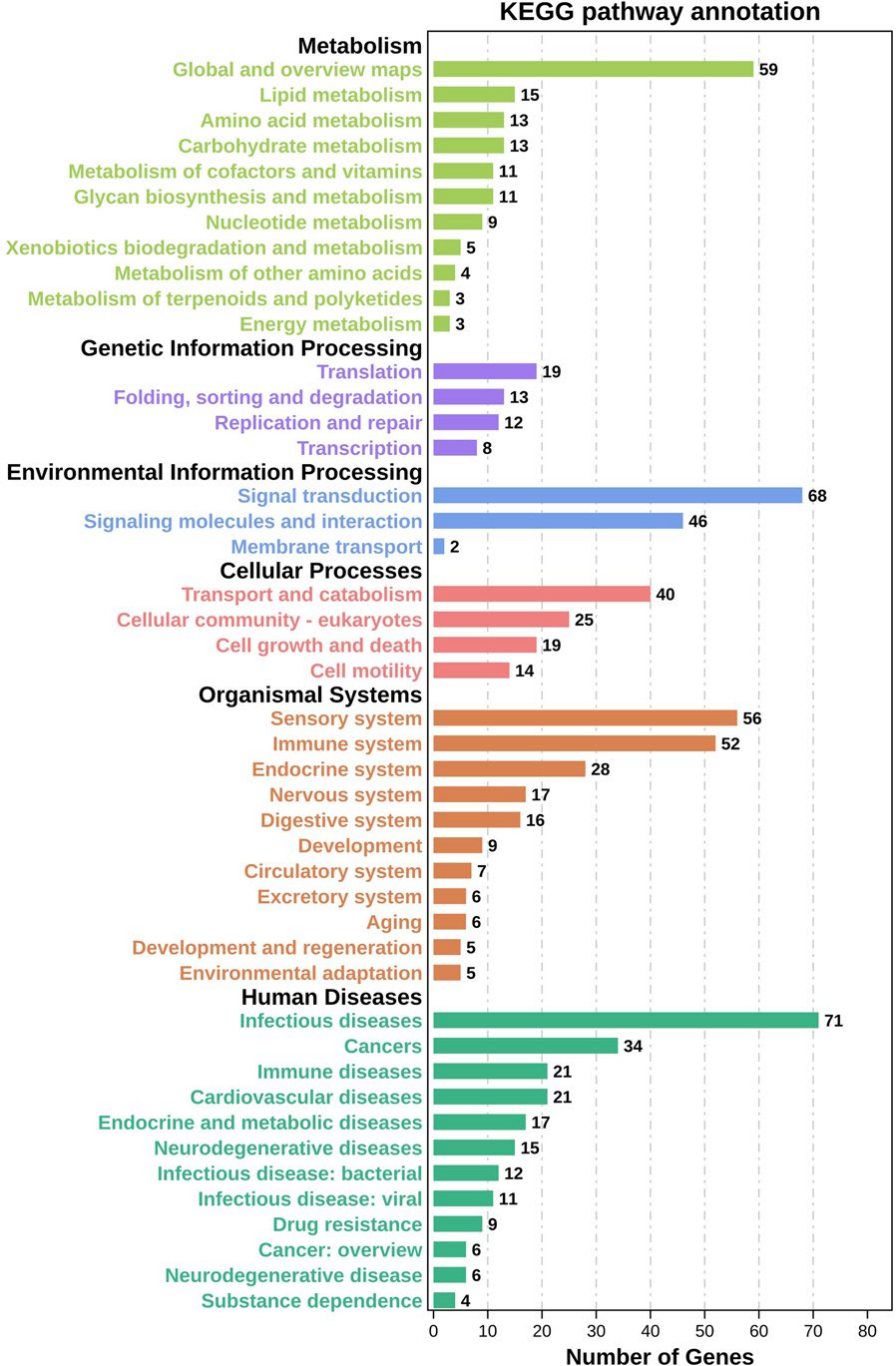
Enrichment pathways, green is Human Disease, orange is organismal systems, pink is cellular processes, blue is environmental information processing, purple is genetic information processing, yellow is metabolism

To further understand the impact of CNV on various aspects of pig growth, this study used the GO database of the DAVID website to perform Gene Ontology enrichment analysis and pathway analysis of the genes in the above 461 CNVRs with Ensemble ID. We first submitted the Ensemble numbers of 461 genes to the Biomart site of the Ensemble website to search for human homologous genes and conducted one-to-one screening to ensure the uniqueness of the homologous genes obtained. We then submitted the obtained homologous gene information to the DAVID website, and GO analysis was performed, as shown in Fig. 3. In Fig. 4, functional clustering included three aspects: molecular biological function (Molecular Function, MF), cell component (Cell Component, CC), and biological process (Biological Process, BP). Using *P* ≤ 0.05 as the threshold to screen each cluster item, genes within CNVRs were enriched in a total of 52 different functional clusters, including 24 different biological processes (BP) functional clusters, 12 cellular component (CC) functional clusters, and 16 molecular biology functional (MF) clusters. The functional clustering of these genes was determined to involve many biological processes, primarily involving cell adhesion, embryonic development, and cell differentiation. These clustering results indicate that the genes in CNVRs may be more involved in behavioral adaptation during the process of growth, development, immunity, and domestication, and those that are beneficial to the organism may be preserved by the evolutionary process.

## Discussion

In this study, the BioMart tool was used to identify genes situated within CNVRs, and DAVID was then used to search the GO terms of genes contained in CNVRs. Combined with the annotation information of the KEGG-GENES database, the purpose of obtaining these results was to speculate on the function of these genes and the possible effects of CNVRs on the biological traits of commercial Chinese native and foreign pig breeds.

The analysis of the gene distribution in CNVRs showed that CNVRs were more concentrated in chromosome segments with lower gene densities. The reason for this may be that CNVs in the genome originate from segmental duplication regions, which often contain redundant sequences with less gene distribution. It is also possible that CNVs in gene-enriched regions are easier to order and create functional genes, and cause damage, which is detrimental to the survival of the organism, so they are eliminated under the action of natural selection.

CNVs can lead to phenotypic variation related to single gene diseases, complex diseases or quantitative traits through molecular mechanisms such as gene structure change, gene fusion and dose effect [13]. For example, a CNV-based whole genome-associated analysis found functional genes related to feed conversion rate and growth and development [14]. The explicit white hair color of the pig is related to the repetition of the 450 kb fragment containing the Kit gene [15]. Mei et al. [16] conducted a genome-wide CNV study on six native cattle breeds in China, and found that a large number of adaptive (*BOLA-DQB* and *EGLN2*) and hair color (*KIT* and *MITF*) copy number variation information.

Existing studies have found that genes in CNVRs are mainly involved in immunity, the sensory perception of the external environment (involving smell, vision, and taste), the response to stimuli, and neurodevelopment, while they are relatively less involved in nucleic acid binding, metabolism, and cell proliferation [17]. This may be because the occurrence of gene CNVs with important functions is the result of strong purification selection. When a CNV occurs in the coding region, if the affected gene has an important role in growth, the occurrence of the variation is highly likely to be harmful to the organism and is therefore quickly eliminated. This is consistent with our analysis results. A total of 798 genes were obtained from 348 CNVRs. GO analysis found that these genes were mainly involved in cell adhesion, phosphorylation, and learning, while a small number of genes were involved in embryonic development and cell differentiation.

Copy numbers have varieties in the animal group. Some unique CNVs may be selected during the domestication, resulting in the species specificity. The independent CNV between different varieties may cause variety differences to differences in variety differences [18]. There are differences in lipid metabolism, fat deposition, and disease resistance between Chinese local pigs and Western commercial pigs. The main reason may be because Chinese pig local groups and European pig groups have different domestication or variety formation processes [19]. Genetic background information [20]. In this study, the resequencing data of AQSW pigs and Duroc pigs were used for CNV detection, and the genome structure of these Chinese and foreign pig populations was studied. AQSW pigs were found to have advantages in stress resistance, fat metabolism, and cell biology, and this finding provides useful information for the development of breeding programs and the understanding of disease resistance for this population.

Through GO annotation and KEGG enrichment analysis, six genes related to fat metabolism, reproductive performance, and stress resistance were found in the CNVRs of the whole genome; namely *DPF3*,* LEPR*, *MAP2K6*, *PPARA*, *TRAF6*, and NLRP4. Among them, the *DPF3* gene located on chromosome 7 is related to the number of nipples in pigs. The number of nipples of a sow is an important indicator of its ability to raise a large number of piglets. In sows, the combination of having a large number of nipples and a large litter size is the physiological basis for achieving high reproductive performance. A lack of teats leads to insufficient colostrum intake in piglets, which negatively affects piglet body weight and immunity, and may also lead to piglet death due to starvation and crushing [21]. It has been reported that sow teat number is highly important for piglet mortality, body weight gain, and uniform growth [22]. Related studies have also found that the number of nipples in pigs varies among breeds [23, 24], and has a strong correlation with reproductive performance.

The *LEPR* gene plays an important role in the decomposition of fatty acids and the maintenance of energy balance in the body. *LEPR* mRNA is expressed in sheep adipocytes as well as in human adipocytes [25]. Numerous studies have shown that *LEPR* plays an important role in regulating body fat deposition [26–28]. The pig *MAP2K6* gene is located on chromosome 12 and is mainly involved in the regulation of reproductive traits [29, 30]. The *PPARA* gene is an important isoform of the *PPARs* family and was the first identified member. As a major transcriptional regulator of fatty acid oxidase, it regulates the normal metabolism of lipids in the body [31].

Both the *NLRP4* and *TRAF6* genes are related to the immune inflammatory response [32]. The TRAF6 gene is a key linker molecule in the regulatory pathways of the tumor necrosis factor (TNF) superfamily, toll-like receptor (TLR) family, and interleukin-1 receptor (TIR) superfamily, as well as an important linker molecule in the innate immune signaling pathway [33, 34].

The above findings indicate that the occurrence of CNVs may have an impact on the immune response, fat metabolism, and reproduction of LB pigs.

## Conclusion

In this study, the resequencing data of AQSW pigs and Duroc pigs were used for CNV detection, and a total of 881 CNVRs were detected in these two populations. The results show that the AQSW and Duroc pig populations have significant differences in the genomic localization of genetic variation. We also identified candidate genes related to lipid metabolism, reproductive traits, and stress resistance; namely *DPF3*, *LEPR*, *MAP2K6*, *PPARA*, *TRAF6*, and *NLRP4*. The results of this study lay a foundation for subsequent studies on potential mutations associated with the observed phenotypic variation.

## Materials and methods

### Quality control

FASTP software version 0.18.0 [35] was used to filter the raw data of the Illumina platform to obtain relatively high-quality sequencing data for clean reads assembly analysis. Our filtering criteria were as follows: (1) removal of reads containing unknown nucleotides (N) ≥ 10%; (2) removal of reads with a Phred quality score ≤ 20 bases ≥ 50%; (3) deletion of reads containing linkers. According to the above criteria, some low-quality reads; such as those containing primers or adapters, those containing non-ATCG bases, and those with an error rate of more than 30%; were removed.

### Comparative analysis

The pig reference genome Sus scrofa 11.1 can be obtained from the Ensemble database, and be used to detection of copy number variation. We used the alignment software BWA [36] with the BWA-MEM algorithm to align the filtered reads to the reference genome, and the alignment parameter was -k 32-M; (Dior Biotechnology Co., Ltd. 1.129). Picard was used to mark the repeated reads and count the depth and coverage of the marked reads. Coverage statistics were performed using BEDTools [37].

### Detection of CNVs and CNVRs

This study used CNVnator [38] to detect the CNVs. We refer to the threshold recommended in the CNVnator methodology article to filter reliable CNVs to facilitate subsequent analysis of gene copy numbers. The employed criteria were as follows: (1) e-value < 0.01; (2) RD < 0.7 in the deletion region; RD > 1.3, CNV interval size > 1 kb. The genome-wide detection of copy number variants in AQ and Duroc pig breeds by whole-genome sequencing of DNA was performed to identify breed copy numbers.

The CNVs in the two populations of pigs were integrated using BEDTools to determine the CNVRs of each population. Using Ensemble (the website), the reference genome of pigs (Sus scrofa 11.1) was used to find genes that overlapped with CNVRs. The screened genes were then further analyzed for functional enrichment by PANTHER [39].

### Gene detection and enrichment analysis

Using the BioMart [40] gene database based on the pig reference genome sequence in Ensemble, the genes located in CNVRs were searched on the basis of having at least 1 bp overlapping, and the functional annotation of the genes in CNVRs was performed.

Gene Ontology terms (GO) and Kyoto Encyclopedia of Genes and Genomes (KEGG) pathway analyses were performed using the DAVID Bioinformatic Database [41].

## Data Availability

This study collected data from 40 pigs, including 20 healthy LB pigs and 20 Duroc pigs. We obtained the re-sequencing data from 20 Duroc pigs using NCBI [7]. We sampled 20 unrelated LB pigs from the LB conservation farm (Anqing, China; longitude, 116° 330 E; latitude, 30° 190 N). Genomic DNA was extracted from the ear samples of pigs using the standard phenol–chloroform method [42], stored at 4 °C to avoid freeze-thawing, and tested for its nucleic acid concentration (as ng/ml) using a Nanodrop instrument. The DNA was subsequently fragmented and treated following the Illumina DNA sample preparation protocol. The protocol employed a process of end-repaired, A-tailed, ligated to paired-end adaptors and PCR amplification with 350-bp inserts. The constructed libraries were then sequenced by the Illumina HiSeq X Ten platform (Illumina, San Diego, CA) for 150-bp paired-end reads at Novogene (Beijing, China).
